# Editorial: Reviews in cardiovascular pharmacology: 2023

**DOI:** 10.3389/fphar.2025.1566159

**Published:** 2025-02-14

**Authors:** Yusof Kamisah, Ismail Laher, Mas Rizky A. A. Syamsunarno

**Affiliations:** ^1^ Department of Pharmacology, Faculty of Medicine Universiti Kebangaan Malaysia, Kuala Lumpur, Malaysia; ^2^ Cardiovascular and Pulmonary University Research Group, Universiti Kebangaan Malaysia, Bangi, Selangor, Malaysia; ^3^ Department of Anesthesiology, Pharmacology and Therapeutics, Faculty of Medicine, University of British Columbia, Vancouver, BC, Canada; ^4^ Department of Biomedical Sciences, Faculty of Medicine, Universitas Padjadjaran, Bandung, Indonesia

**Keywords:** cardiac, vascular, fibrosis, atherosclerosis, inflammation, oxidative stress

## 1 Introduction

Cardiovascular disease remains a leading cause of death globally, accounting for approximately 17.9 million fatalities in 2019 (World Health Organization, 2021). Cardiovascular diseases include hypertension, atherosclerosis, ischemic heart disease, stroke and heart failure, and represent a significant socioeconomic burden. The Research Topic *“Reviews in Cardiovascular Pharmacology: 2023*”provides an overview of both the pathogenesis and pharmacological advances in cardiovascular diseases. This Research Topic features 12 articles, each offering in-depth discussions of recent findings on the mechanisms underlying cardiovascular diseases, along with the development and application of novel cardiovascular therapies. The articles cover a wide range of topics that include recent advancements in clinical trials, emerging concepts in drug mechanisms, therapeutic strategies, and challenges related to pharmacokinetics.

Nitric oxide (NO) is a highly reactive gaseous molecule released by endothelial cells in blood vessels that plays a crucial role in mediating protective cardiovascular effects, for instance vasodilation (Siti et al., 2019). Impaired NO function often occurs before the clinical onset of cardiovascular (Abd-Elmoniem et al., 2024) disease. This endogenous vasodilator binds to soluble guanylate cyclase (sGC), stimulating the synthesis of cyclic guanosine monophosphate (cGMP). Elevated cGMP activates protein kinase G (PKG), which lowers intracellular calcium levels in vascular smooth muscle cells and induces vasodilation (Mishra et al., 2025). Yin et al. reviewed the progress of guanylate cyclase activators to stimulate the NO-sGC-cGMP signaling pathway in patients with cardiovascular disease. These include riociguat, vericiguat, praliciguat, olinciguat, cinaciguat, ataciguat, runcaciguat, mosliciguat, and BI 685509, the latter still undergoing clinical trials. Therapies such as riociguat and vericiguat show benefits such as improved quality of life in patients with heart failure and pulmonary hypertension, although definitive conclusions for other agents are not yet available. Additionally, Yin et al. discussed the pharmacology of guanylate cyclase-C (GC-C) agonists (linaclotide and plecanatide) for the treatment of digestive conditions. These agents improve symptoms of irritable bowel syndrome with constipation (IBS-C) by activating the GC-C/cGMP pathway, reducing submucosal afferent neuron excitation and reducing abdominal pain.

While guanylate cyclase mediates its effects primarily through cGMP to cause vasodilation and smooth muscle relaxation (Siti et al., 2015), adenylyl cyclase produces cAMP to regulate diverse functions, such as metabolism, heart rate, and neurotransmitter release (Marsden and Dessauer 2019). Adenylyl cyclase isoforms 5 (AC5) and 6 (AC6) are highly expressed in cardiac tissues, where they are important in translating signals from β-adrenergic receptors into intracellular cAMP production (Marsden and Dessauer 2019). Maghsoudi et al. highlighted the distinct regulatory mechanisms and physiological roles of these isoforms, particularly in calcium handling, myocardial contractility, and adaptive responses to catecholamine stimulation. AC5 plays a key role in cardiac and vascular function, and its inhibition has cardioprotective effects. In contrast, AC6 plays a significant role in vasodilation and is particularly enriched in neonatal tissues, with its overexpression enhancing cardiac repair. The potential of targeting these isoforms for therapeutic intervention, especially in conditions such as heart failure where dysregulated β-adrenergic signaling contributes to disease progression, is also discussed.

Atherosclerosis is a significant underlying cause of cardiovascular disease. It is a chronic, progressive condition marked by the accumulation of plaques composed of lipids, cholesterol, calcium, and cellular debris within the walls of arteries (Wang et al., 2021). Inclisiran is a novel cholesterol-lowering drug with promising pharmacological properties, as described by Zhang et al. The drug utilizes small interfering RNA (siRNA) to silence the expression of the proprotein convertase subtilisin/kexin type 9 (PCSK9) gene, which encodes a protein responsible for degrading low-density lipoprotein (LDL) receptors in the liver. By inhibiting PCSK9 expression, inclisiran increases the availability of LDL receptors on hepatic cells, thereby enhancing the clearance of LDL cholesterol from the bloodstream. This leads to a sustained reduction in LDL cholesterol levels, as demonstrated in clinical trials, thereby reducing the risk of atherosclerosis progression.

Inflammation is a key contributor to the pathogenesis of many cardiovascular diseases such as atherosclerosis. The NOD-like receptor protein 3 (NLRP3) inflammasome is an inflammatory mediator activated in cardiovascular diseases. Upon activation, NLRP3 triggers pyroptosis, a form of programmed cell death characterized by inflammatory cell death and the release of pro-inflammatory cytokines (Zhang et al., 2023). Ding et al. comprehensively discussed the inhibitory effects of exercise on NLRP3 expression and pyroptosis in atherosclerosis, obesity, diabetic cardiomyopathy, myocardial infarction, hypertension, and heart failure. They demonstrated that NLRP3 plays a critical role in the pathogenesis of cardiovascular disease. In addition to these molecular mechanisms, microbial infections have also been implicated in cardiovascular disease. Aramouni et al. suggested that *Helicobacter pylori* infection may contribute to atherogenesis by promoting foam cell formation and triggering a chronic immune response. These findings underscore the multifaceted nature of inflammation in cardiovascular disease, spanning both molecular and infectious contributions.

Traditional Chinese Medicine (TCM) has emerged as a significant source of alternative and complementary therapies, offering bioactive compounds with therapeutic potential against various diseases. Bioactive monomers extracted from TCM, such as geniposide, astragaloside IV, genkwanin, and tanshinone IIA, target non-coding RNAs to alleviate pathological processes associated with atherosclerosis. These non-coding RNAs modulate mechanisms underlying atherosclerosis, for instance inflammation, oxidative stress, adipogenesis, apoptosis, and autophagy (Liu et al.). In addition to their role in atherosclerosis, non-coding RNAs are also implicated in broader cardiovascular diseases. Active monomers such as quercetin, tripterine, notoginsenoside R1, and berberine—also derived from TCM—reduce myocardial fibrosis effects by modulating non-coding RNAs (Wang et al.). These findings emphasize the broad therapeutic potential of targeting non-coding RNAs in the treatment of various cardiovascular conditions.

In the context of cardiovascular disease, Shenfu, a TCM formulation derived from ginseng (*Panax ginseng* C.A. Mey) and aconite (*Aconitum carmichaelii* Debeaux), may have therapeutic benefits. Injecting Shenfu improves cardiac function in patients diagnosed with myocardial infarction, cardiac arrest following resuscitation, and heart failure (Xu et al.). In animal models of cardiovascular disease, it attenuates oxidative stress and inflammation by downregulating the nuclear factor kappa-light-chain-enhancer of activated B cells (NF-κB) signaling pathway. Additionally, it reduces apoptosis and fibrosis by modulating the transforming growth factor-beta (TGF-β)/Smads signaling pathway. Moreover, ginsenoside Rg3, extracted from *P. ginseng*, has shown promising effects in mitigating heart failure by reducing inflammation, oxidative stress, apoptosis, and fibrosis in various animal models of heart disease and mental illness (Shi et al.).

Zhang et al. conducted a comprehensive review on amentoflavone, a flavonoid with pharmacological potential for treating neurological disorders and cardiocerebrovascular diseases by mitigating inflammation, oxidative stress, and reducing lipid levels. They highlight the challenges in improving its pharmacokinetic profile due to its low solubility and poor oral bioavailability. The study by Wang et al. examined the challenges associated with tyrosine kinase inhibitors, a class of tumor-targeted therapies that includes apatinib. These drugs can cause microcirculatory rarefaction that impairs microvascular growth. The authors hypothesized that inhibiting the Notch signaling pathway may mitigate the microvascular changes induced by tyrosine kinase inhibitors.

Heat stroke can cause myocardial injury by disrupting electrolytes, particularly those associated with the sodium-potassium pump (Wang et al., 2019). Xia et al. explored the pathogenesis of heat stroke-induced myocardial injury, highlighting hypercytokinemia as a result of increased inflammation, metabolic abnormalities, and protein dysregulation. These changes contribute to endothelial dysfunction, circulatory shock, and ultimately cardiomyocyte death. The article also discusses several strategies for the management of heat stroke.

In conclusion, this Research Topic presents a series of articles that explore various aspects of cardiovascular health. It provides valuable insights into the molecular mechanisms underlying the pathogenesis of cardiovascular disease along with the protective effects of different cardiovascular drugs. Collectively, these articles serve as a vital resource for researchers, clinicians, and healthcare professionals striving to improve patient outcomes in cardiovascular care.
